# Gene set enrichment analysis identifies immune subtypes of kidney renal clear cell carcinoma with significantly different molecular and clinical properties

**DOI:** 10.3389/fimmu.2023.1191365

**Published:** 2023-06-23

**Authors:** Zuobing Chen, Wenxiu Cao, Jiangti Luo, Zeinab Abdelrahman, Qiqi Lu, Huafen Wang, Xiaosheng Wang

**Affiliations:** ^1^ Department of Rehabilitation Medicine, The First Affiliated Hospital, Zhejiang University School of Medicine, Hangzhou, China; ^2^ Biomedical Informatics Research Lab, School of Basic Medicine and Clinical Pharmacy, China Pharmaceutical University, Nanjing, China; ^3^ Cancer Genomics Research Center, School of Basic Medicine and Clinical Pharmacy, China Pharmaceutical University, Nanjing, China; ^4^ Big Data Research Institute, China Pharmaceutical University, Nanjing, China; ^5^ Centre for Public Health, Queen’s University of Belfast, Belfast, United Kingdom; ^6^ Department of Nursing, The First Affiliated Hospital, Zhejiang University School of Medicine, Hangzhou, China

**Keywords:** kidney renal clear cell carcinoma, tumor heterogeneity, immunological classification, machine learning, multi-omics analysis

## Abstract

**Background:**

Kidney renal clear cell carcinoma (KIRC) is the most prevalent renal malignancy, marked by a high abundance of tumor-infiltrating lymphocytes (TILs) and an unfavorable prognosis upon metastasis. Numerous studies have demonstrated that KIRC possesses a tumor microenvironment that is highly heterogeneous, and this is associated with significant variations in the effectiveness of most first-line drugs administered to KIRC patients. Therefore, it is crucial to classify KIRC based on the tumor microenvironment, although these subtyping techniques are still inadequate.

**Methods:**

By applying gene set enrichment scores of 28 immune signatures, we conducted a hierarchical clustering of KIRC and determined its immune subtypes. In addition, we conducted a comprehensive exploration of the molecular and clinical features of these subtypes, including survival prognosis, proliferation, stemness, angiogenesis, tumor microenvironment, genome instability, intratumor heterogeneity, and pathway enrichment.

**Results:**

Through cluster analysis, two immune subtypes of KIRC were identified and termed Immunity-High (Immunity-H) and Immunity-Low (Immunity-L). This clustering outcome was consistent in four independent KIRC cohorts. The subtype Immunity-H exhibited elevated levels of TILs, tumor aneuploidy, homologous recombination deficiency, stemness, and proliferation potential, along with a poorer prognosis for survival. Despite this, the Immunity-L subtype demonstrated elevated intratumor heterogeneity and a stronger angiogenesis signature in contrast to Immunity-H. According to the results of pathway enrichment analysis, the Immunity-H subtype was found to be highly enriched in immunological, oncogenic, and metabolic pathways, whereas the Immunity-L subtype was highly enriched in angiogenic, neuroactive ligand-receptor interaction, and PPAR pathways.

**Conclusions:**

Based on the enrichment of immune signatures in the tumor microenvironment, KIRC can be categorized into two immune subtypes. The two subtypes demonstrate considerably distinct molecular and clinical features. In KIRC, an increase in immune infiltration is linked to a poor prognosis. Patients with Immunity-H KIRC may exhibit active responses to PPAR and immune checkpoint inhibitors, whereas patients with Immunity-L may manifest favorable responses to anti-angiogenic agents and immune checkpoint inhibitors. The immunological classification provides molecular insights into KIRC immunity, as well as clinical implications for the management of this disease.

## Introduction

Kidney renal clear cell carcinoma (KIRC) is the most frequently encountered histologic subtype of renal cell carcinoma, constituting roughly 75% of all renal cell carcinomas (1). The main approach for treating patients with stage I-III KIRC is surgical treatment. Nevertheless, the recurrence takes place in over 30% of KIRC patients following partial or complete nephrectomy (2). Furthermore, an unfavorable prognosis is frequently observed in patients with metastatic KIRC (3). Immune checkpoint blockade (ICB) has recently exhibited remarkable therapeutic effectiveness in several forms of cancer, such as melanoma (4), non-small cell lung cancer (5), and head and neck cancer (6). It is noteworthy that despite the fact that KIRC does not possess the usual hallmark of most immunotherapy-responsive cancers, i.e., high tumor mutation burden (TMB), ICB treatment has been shown to achieve high response rates in the treatment of advanced KIRC (7).

Targeted therapy, immunotherapy, or a combination of the two are the foremost treatment options for metastatic KIRCs (8). Despite this, the current treatment methods are effective only for a subset of KIRC patients (9). Accordingly, there is an urgent necessity to optimize immunotherapeutic and targeted therapeutic strategies for KIRC patients. A significant hurdle to the clinical treatment of KIRC is posed by the highly heterogeneous tumor microenvironment, as demonstrated by various studies (10, 11). Several parameters have been examined to anticipate treatment responses among KIRC patients, including mismatch repair defects (12), systemic immunoinflammatory index (13), Fuhrman grading (14), and levels of tumor-infiltrating lymphocytes (TILs) (15). Most solid tumors have a significant association between immunotherapy responses and TMB, PD-L1 expression, and TILs levels (16–18). Nevertheless, these parameters may not be applicable to KIRC as this form of cancer is typically characterized by low TMB (19). Furthermore, the CheckMate025 trial has demonstrated that response rates to the Nabumab monotherapy against PD-1 does not correlate with PD-1 expression (20). Despite the negative association of CD8+ T cells’ abundance with survival prognosis in KIRC patients (15), the high level of TILs could indicate that KIRC is a feasible candidate for immunotherapy. Thus, the subtyping of KIRC based on the immune microenvironment could potentially have significant implications for the diagnosis, prognosis, and treatment of this disease. To this end, we conducted a clustering analysis of KIRC based on the enrichment levels of 28 immune signatures and identified its immune subtypes. We conducted additional analysis on a range of molecular and clinical features associated with these subtypes. It is expected that the immune-specific subtyping of KIRC will provide valuable insights into the biology of cancer and clinical implications for its management.

## Methods

### Datasets

Gene expression profiles of 537 KIRC patients were downloaded from The Cancer Genome Atlas (TCGA), along with their clinical data from the cBioPortal for Cancer Genomics (https://www.cbioportal.org/). From the genomic data commons (GDC) data portal (https://portal.gdc.cancer.gov/), we downloaded profiles of somatic mutations (“maf” file) and somatic copy number alterations (SCNAs) (“SNP6” files) for the 537 TCGA-KIRC patients. Furthermore, we acquired gene expression profiles and clinical data for three KIRC cohorts from the NCBI gene expression omnibus (GEO) (https://www.ncbi.nlm.nih.gov/geo/), with accession ID GSE29609, GSE40435, and GSE73731. The datasets are summarized in **Supplementary Table S1**. Prior to performing subsequent analyses, we conducted normalization of all RNA sequencing (RNA-seq) gene expression values through log2(TPM+1) transformation.

### Gene set enrichment analysis

We used the single-sample gene set enrichment analysis (ssGSEA) (21, 22) to score the enrichment levels of immune signatures, biological processes, and DNA repair pathways, including 28 immune cell types, exhausted CD8+ T cells, proliferation, stemness, angiogenesis, and pathways of mismatch repair, base excision repair, homology-dependent recombination, and DNA replication, based on the expression profiles of their marker or pathway genes. The marker genes of the immune signatures and biological processes were obtained from several publications (23–27), and the DNA repair pathways’ genes were downloaded from KEGG (28), as shown in **Supplementary Table S2**.

### Clustering analysis

We hierarchically clustered KIRC to identify its immune subtypes based on the enrichment scores of 28 immune cell types. These immune cells included CD56^bright^ natural killer (NK) cells, effector memory CD4 T cells, eosinophil, CD56^dim^ NK cells, type 17 T helper cells, activated B cells, monocytes, memory B cells, activated CD4 T cells, type 2 T helper cells, plasmacytoid dendritic cells, neutrophils, macrophages, effector memory CD8 T cells, myeloid-derived suppressor cells (MDSC), immature B cells, T follicular helper cells, NK cells, immature dendritic cells, mast cells, type 1 T helper cells, activated dendritic cells, central memory CD4 T cells, gamma delta T cells, central memory CD8 T cells, regulatory T cells, activated CD8 T cells, and NK T cells (27). Before clustering, we normalized the ssGSEA scores by z-score and transformed them into distance matrices by the R function “dist” with the parameter method  ”Euclidean.” We performed hierarchical clustering using the function “hclust” in the R package “Stats” with the parameters: method = ”ward.D2” and members = NULL.

### Principal component analysis

We performed PCA of the TCGA-KIRC cohort based on their ssGSEA scores of the 28 immune signatures and the immune subtype labels of the samples. This analysis was performed with the R package “FactoMineR” to downscale the 28 features into two principal components.

### Evaluation of immune cell infiltration levels, tumor purity, and stromal content in KIRC

The ESTIMATE algorithm (29) was used to evaluate the immune cell infiltration levels (immune scores), tumor purity, and stromal content (stromal scores) for each KIRC sample. ESTIMATE (29) assesses these parameters based on the expression profiles of associated genes.

### Survival analysis

We utilized the Kaplan–Meier method (30) to compare the survival time between different groups of cancer patients. Kaplan–Meier curves were plotted to show the survival rates. A total of two survival endpoints were analyzed, including overall survival (OS) and disease-free survival (DFS). The log-rank test was used to evaluate the significance of survival time differences with a threshold of *P* < 0.05. The survival analyses were performed in the TCGA-KIRC and GSE29609 datasets, where related data were available.

### Evaluation of TMB, tumor aneuploidy, and homologous recombination deficiency

A tumor’s TMB was defined as its total count of somatic mutations. We employed the ABSOLUTE algorithm (31) to assess the ploidy score representing the tumor aneuploidy level for each TCGA-KIRC sample, with the input of “SNP6” files. The HRD scores in 9,125 TCGA cancer samples were defined based on HRD loss of heterozygosity, large-scale state transitions, and the number of telomeric allelic imbalances (32). We extracted the results of HRD scores in TCGA-KIRC from the data.

### Evaluation of intratumor heterogeneity in KIRC

The ITH levels were evaluated by the DITHER algorithm (33), which measures ITH based on the entropy of alterations in somatic mutations and copy numbers in tumors.

### Pathway analysis

We performed pathway enrichment analysis to identify KEGG pathways enriched in the KIRC immune subtypes by GSEA (34) with a threshold of adjusted *P*-values (false discovery rate (FDR)) < 0.05. GSEA output the enriched pathways in an immune subtype based on the input of the significantly upregulated genes in this subtype versus another subtype. The significantly upregulated genes were identified using a threshold of two-tailed Student’s *t* test FDR < 0.05 and fold change of mean gene expression levels > 1.5. In addition, we used the weighted gene co-expression network analysis (WGCNA) (35) to identify gene modules and significantly related GO traits enriched in the subtypes. The WGCNA analysis was carried out using the R package “WGCNA” (version 1.68).

### Class prediction

We predicted the KIRC immune subtypes based on the ssGSEA scores of the immune signatures by the Random Forest (RF) algorithm (36). The number of trees in the RF was set as 500, and the predictors were the 28 immune signatures. The accuracy and weighted F-score were reported as the prediction performance. The RF algorithm was implemented with the “randomForest” R package.

### Statistical analysis

In comparisons of two classes of normally-distributed data, such as gene expression levels, we used the two-tailed Student’s *t* test. We used the one-tailed Mann- Whitney *U* test to compare two classes of non-normally distributed data, such as TIL levels in pathological sections, ssGSEA scores, ITH scores, TMB, and HRD scores. Fisher’s exact test was utilized to analyze the association between two categorical variables. To adjust for *P*-values in multiple tests, FDRs were calculated using the Benjamini-Hochberg method (37). We performed all statistical analyses with the R programming language (version 3.6.1).

## Results

### Clustering analysis identifies two immune subtypes of KIRC

**Figure 1:**
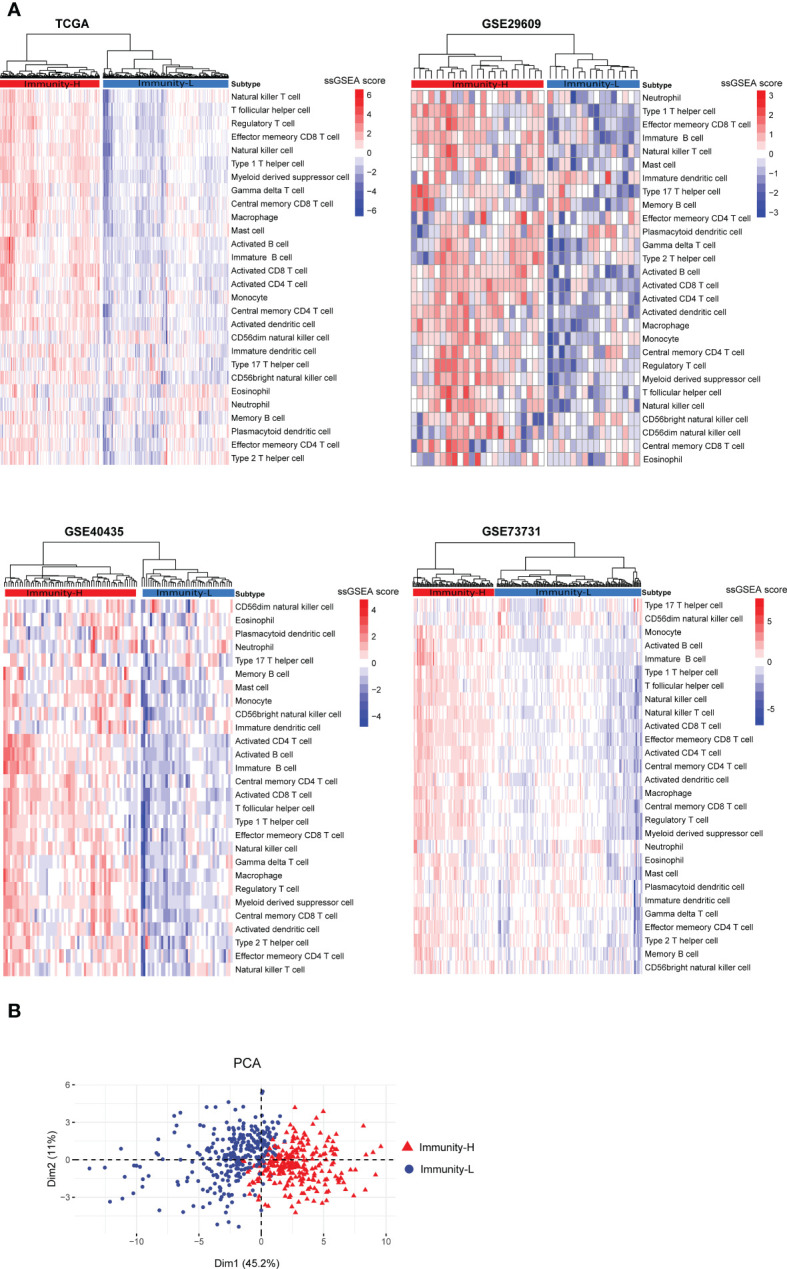
Clustering analysis identifies two immune subtypes of KIRC based on transcriptomic profiles. **(A)** Based on the enrichment scores of 28 immune cell types, hierarchical clustering uncovers two immune subtypes of KIRC (Immunity-H and Immunity-L), consistently in four transcriptome datasets. **(B)** Principal component analysis (PCA) shows both subtypes to be clearly distinguished based on the enrichment scores of the 28 immune signatures in the TCGA-KIRC cohort.

Based on the enrichment scores of 28 immune cell types (27), hierarchical clustering identified two subtypes of KIRC consistently in the four transcriptome datasets (TCGA-KIRC, GSE29609, GSE40435, and GSE73731) (**Figure 1A**). The two subtypes showed high and low enrichment scores of these immune cells, termed Immunity-H and Immunity-L, respectively. These consistent clustering results demonstrated the reproducibility of this subtyping method. Furthermore, we performed PCA of the TCGA-KIRC cohort based on their ssGSEA scores of the 28 immune signatures and confirmed both subtypes to be clearly distinguished (**Figure 1B**).

**Figure 2:**
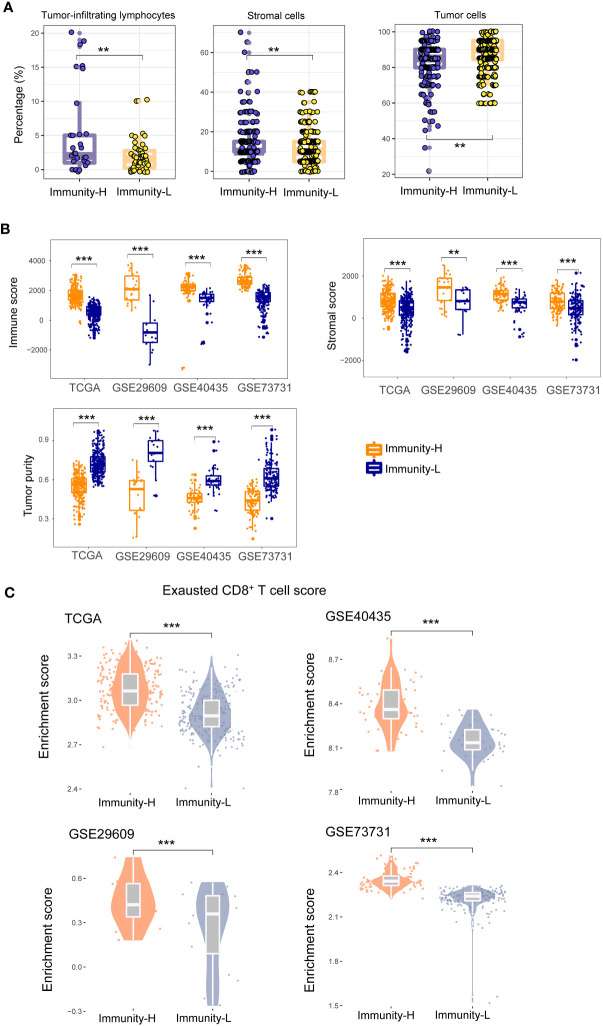
Comparisons of the enrichment of tumor-infiltrating lymphocytes (TILs) and stromal cells and tumor cells between the immune subtypes of KIRC. **(A)** Pathological slides data for TCGA-KIRC show that the percentages of TILs and percentages of stromal cells are significantly higher in Immunity-H than in Immunity-L KIRCs, and the percentages of tumor cells are significantly lower in Immunity-H than in Immunity-L KIRCs. **(B)** The immune scores and stromal scores by ESTIMATE (29) are significantly higher in Immunity-H than in Immunity-L KIRCs, and tumor purity is significantly lower in Immunity-H than in Immunity-L KIRCs, in all four KIRC cohorts. **(C)** The abundance (enrichment scores) of exhausted CD8+ T cells is significantly higher in Immunity-H than in Immunity-L KIRCs in all four KIRC cohorts. The one-tailed Mann–Whitney *U* test *P*-values are shown. *P < 0.05, **P < 0.01, ***P < 0.001, ^ns^ *P* ≥ 0.05; it also applies to the following figures.

In TCGA-KIRC, the pathological slides data confirmed that the percentages of TILs were significantly higher in Immunity-H than in Immunity-L KIRCs (*P* = 0.01); the percentages of stromal cells were also higher in Immunity-H than in Immunity-L KIRCs (*P* = 0.007); however, the percentages of tumor cells were lower in Immunity-H than in Immunity-L KIRCs (*P* = 0.007) (**Figure 2A**). As expected, the immune scores and stromal scores by ESTIMATE (29) were significantly higher in Immunity-H versus Immunity-L KIRCs in all four datasets (*P* < 0.01) (**Figure 2B**). By contrast, tumor purity was significantly lower in Immunity-H than in Immunity-L KIRCs. Interestingly, we observed that the abundance of exhausted CD8+ T cells was significantly higher in Immunity-H than in Immunity-L KIRCs, consistently in the four datasets (*P* < 0.05) (**Figure 2C**).

### The immune subtypes of KIRC have significantly different clinical and molecular properties

**Figure 3:**
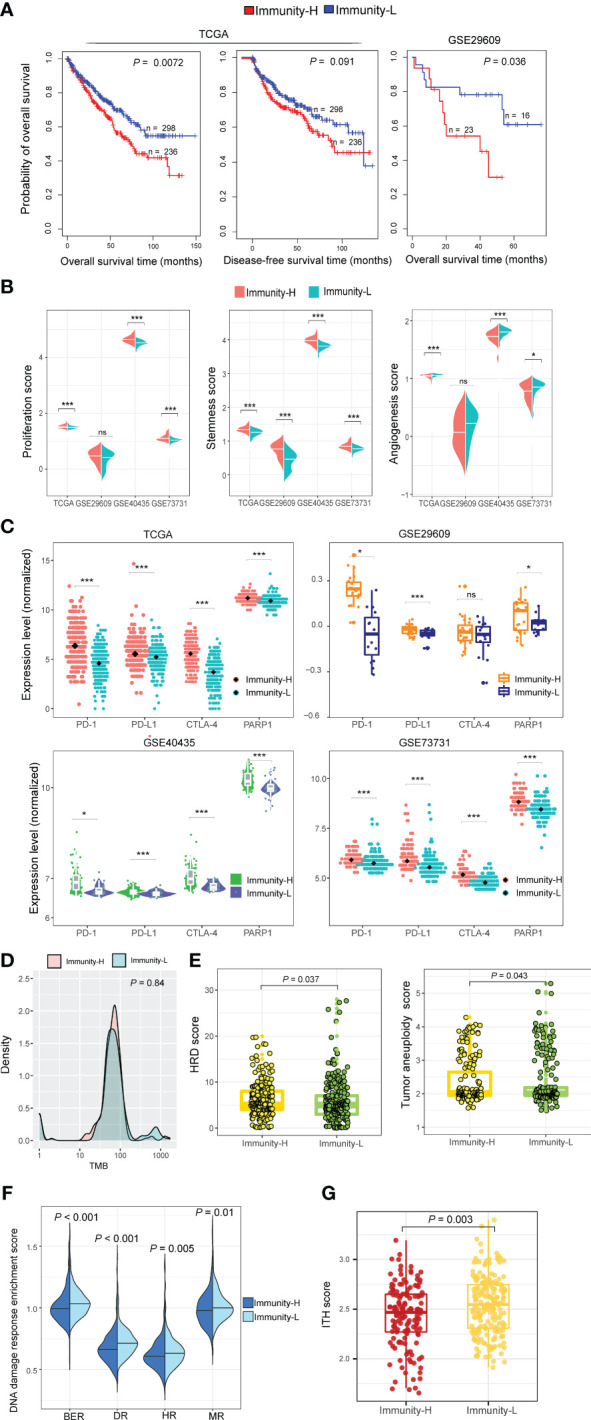
Comparisons of clinical and molecular properties between the immune subtypes of KIRC. **(A)** Kaplan–Meier curves show that Immunity-H patients have significantly worse overall survival than Immunity-L patients in two KIRC cohorts and that Immunity-H patients likely have worse disease-free survival than Immunity-L patients in TCGA-KIRC. The log-rank test *P*-values are shown. **(B)** The scores of proliferation and stemness are higher in Immunity-H than in Immunity-L KIRCs, while angiogenesis scores are lower in Immunity-H than in Immunity-L KIRCs. **(C)** The expression levels of *PD-1*, *PD-L1*, *CTLA-4*, and *PARP1* are higher in Immunity-H than in Immunity-L KIRCs. **(D)** Tumor mutation burden (TMB) shows no significant difference between Immunity-H and Immunity-L KIRCs in TCGA-KIRC. **(E)** Tumor aneuploidy levels and homologous recombination deficiency (HRD) scores are significantly higher in Immunity-H than in Immunity-L KIRCs. **(F)** Four DNA repair pathways are significantly upregulated in Immunity-L versus Immunity-H KIRCs. BER: base excision repair; DR: DNA replication; HR: homology-dependent recombination; MR: mismatch repair. **(G)** Immunity-H KIRCs have significantly lower intratumor heterogeneity (ITH) than Immunity-L KIRCs. The one-tailed Mann–Whitney *U* test *P*-values are shown in **(B, D–G)**, and two-tailed Student’s *t* test *P*-values are shown in **(C)**.

In the datasets (TCGA-KIRC and GSE29609) with survival data available, Immunity-H patients displayed significantly worse OS than Immunity-L patients, consistently in both datasets (*P* < 0.05) (**Figure 3A**). Moreover, in TCGA-KIRC, Immunity-H patients likely had worse DFS than Immunity-L patients (*P* = 0.091) (**Figure 3A**). We further compared several cancer-associated phenotypic or molecular features between both subtypes based on transcriptomic profiles, including proliferation, stemness, and angiogenesis. Notably, the scores of proliferation and stemness were likely to be higher in Immunity-H than in Immunity-L KIRCs. In contrast, angiogenesis scores were significantly higher in Immunity-L KIRCs compared to Immunity-H KIRCs (**Figure 3B**). Notably, the expression levels of *PD-1*, *PD-L1*, *CTLA-4*, and *PARP1*, which are targets of immunotherapy or targeted therapy, were significantly higher in Immunity-H than in Immunity-L KIRCs (*P* < 0.05) (**Figure 3C**). These results suggest that Immunity-H patients are likely more susceptible to ICB and PARP1 inhibitors.

TMB showed no significant difference between both immune subtypes of TCGA-KIRC (*P* = 0.84) (**Figure 3D**). However, tumor aneuploidy levels were significantly higher in Immunity-H than in Immunity-L KIRCs (*P* = 0.043). HRD scores were also significantly higher in Immunity-H than in Immunity-L KIRCs (*P* = 0.037) (**Figure 3E**). In addition, we found four DNA repair pathways to be significantly upregulated in Immunity-L versus Immunity-H patients, including mismatch repair, base excision repair, homology-dependent recombination, and DNA replication (*P* < 0.05) (**Figure 3F**). Altogether, these results suggest a higher degree of genomic instability in Immunity-H versus Immunity-L patients. Interestingly, Immunity-H KIRCs had significantly lower ITH than Immunity-L KIRCs (*P* = 0.003) (**Figure 3G**), in line with the negative association between ITH and antitumor immune responses (33).

### Identifying pathways and GO highly enriched in the immune subtypes of KIRC

**Figure 4:**
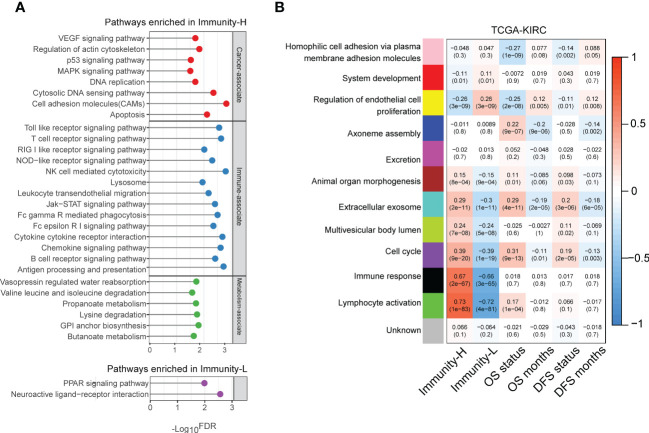
Pathways enriched in the immune subtypes of KIRC. **(A)** KEGG pathways highly enriched in the immune subtypes of TCGA-KIRC identified by GSEA (34). **(B)** Gene modules significantly differentiating KIRC by immune subtypes, survival time, or survival status identified by WGCNA (35) in TCGA-KIRC. The correlation coefficients and *P*-values in parenthesis are shown.

Pathway analysis by GSEA (34) identified numerous KEGG pathways highly enriched in the Immunity-H subtype of TCGA-KIRC. These pathways were mainly involved in oncogenic, immune, and metabolic pathways (**Figure 4A**). The cancer-related pathways included VEGF signaling, p53 signaling, MAPK signaling, DNA replication, and apoptosis pathways. The immune-related pathways included NK cell-mediated cytotoxicity, lysosomes, cytokine receptor interactions, antigen processing and presentation, T cell receptor signaling, chemokine signaling, Toll-like receptor signaling, Fc gamma R-mediated phagocytosis, B-cell receptor signaling, Jak-STAT signaling, Fc-epsilon receptor signaling, NOD-like receptor signaling, leukocyte transendothelial migration, and RIG I-like receptor signaling, cell adhesion molecules (CAMs), and cytoplasmic DNA sensing pathways. The metabolic pathways included valine, leucine, and isoleucine degradation, lysine degradation, methyl butyrate and propionate metabolism, glycosylphosphatidylinositol-anchor biosynthesis, and vasopressin-regulated water reabsorption. These results confirmed the high tumor immunity and genomic instability in the Immunity-H subtype and suggested the activities of these oncogenic and metabolic pathways to be positively associated with KIRC immunity. Pathway analysis also identified two pathways highly enriched in the Immunity-L subtype of TCGA-KIRC, including neuroactive ligand-receptor interaction and PPAR pathways (**Figure 4A**). The PPAR pathway is involved in the regulation of tumor angiogenesis (38), consistent with the strong angiogenic signature shown in Immunity-L KIRCs.

We performed a weighted gene co-expression network analysis of the TCGA-KIRC dataset by WGCNA (35). This analysis identified a set of gene modules that significantly differentiated KIRC by immune subtypes, survival time, or survival status (**Figure 4B**). As expected, the gene modules associated with immune response and lymphocyte activity were highly enriched in the Immunity-H subtype (*r* > 0.65). Moreover, the “lymphocyte activity” module was positively correlated with the OS status (*r* = 0.17), consistent with the negative correlation between immune responses and survival outcomes in KIRC. The “cell cycle” module was also upregulated in Immunity-H KIRCs but downregulated in Immunity-L KIRCs. It agrees with the previous result that Immunity-H KIRCs have a stronger proliferation potential than Immunity-L KIRCs. Notably, this gene module was consistently and negatively correlated with OS and DFS prognosis (**Figure 4B**). Again, it conforms to the worse survival prognosis in Immunity-H than in Immunity-L KIRCs. Like the “cell cycle” module, the “extracellular exosome” module was upregulated in the Immunity-H subtype and negatively correlated with OS and DFS. In contrast, the “endothelial cell proliferation regulation” module was significantly upregulated in the Immunity-L subtype and positively correlated with survival prognosis. It indicates that enhanced immunity in KIRC is associated with impaired endothelial cell proliferation.

### Prediction of the KIRC immune subtypes

**Figure 5:**
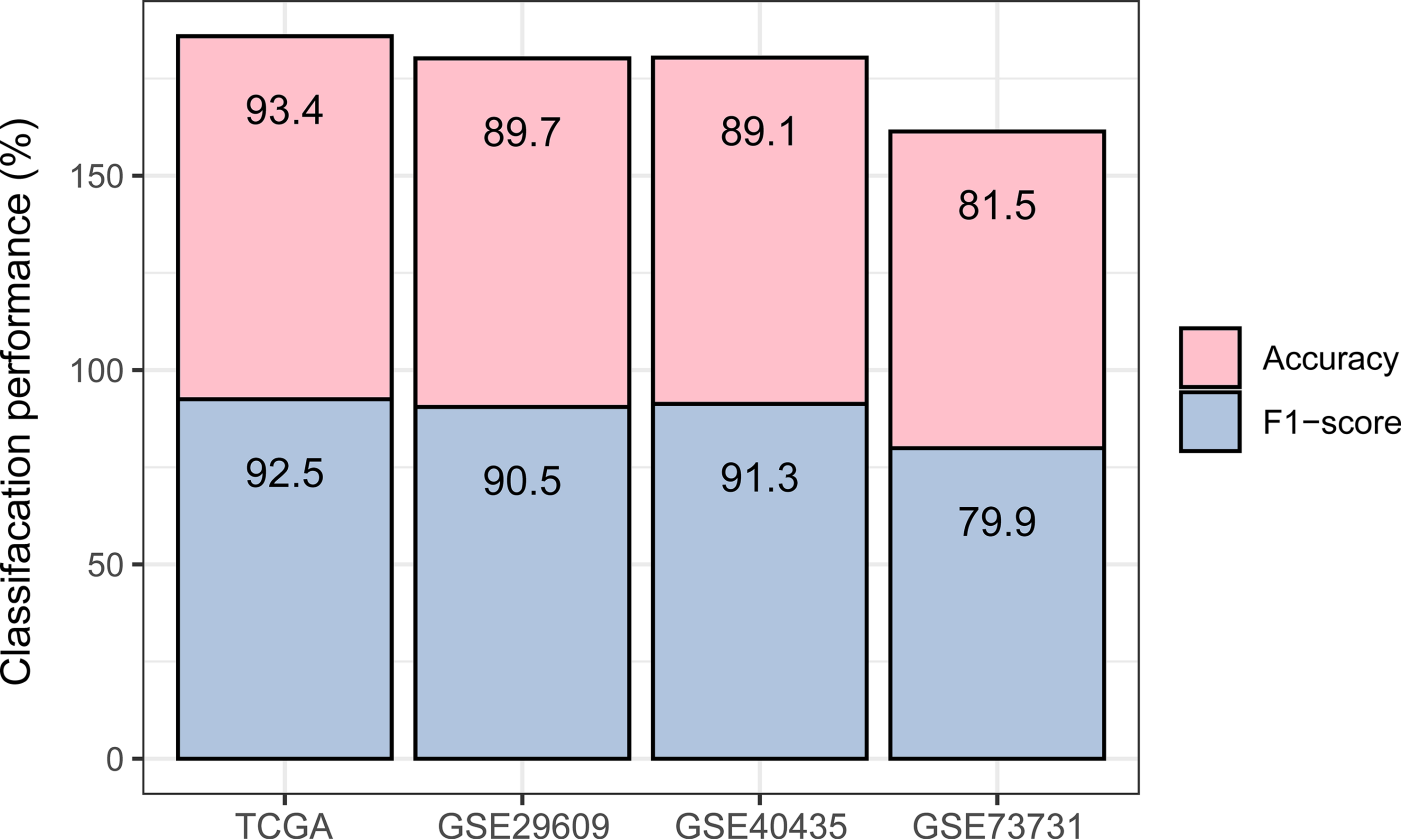
Prediction of the two immune subtypes of KIRC by Random Forest based on the enrichment scores of 28 immune cell types. The 10-fold cross-validation results in the training set and prediction results in the other datasets are shown.

To demonstrate the predictability of the immune subtyping method, we predicted the KIRC immune subtypes based on the enrichment scores of the 28 immune cell types using the RF algorithm (36). Using TCGA-KIRC as the training set, its 10-fold cross-validation (CV) accuracy was 93.4%, and the prediction accuracy was 89.7%, 89.1%, and 81.5% in GSE29609, GSE40435, and GSE73731, respectively (**Figure 5**). The weighted average F-score in these predictions were 92.5%, 90.5%, 91.3%, and 79.9% for TCGA-KIRC, GSE29609, GSE40435, and GSE73731, respectively (**Figure 5**). These results imply the predictability of the immune subtyping method.

## Discussion

The present study introduces a KIRC subtyping approach that relies on the enrichment of 28 immune signatures in the tumor microenvironment. The analysis has successfully identified two immune subtypes of KIRC- Immunity-H and Immunity-L, which have been reproducible in four independent KIRC cohorts. In contrast to Immunity-L, the Immunity-H subtype showed a greater level of TILs, genomic instability, stemness, and proliferation potential, as well as increased enrichment of immunological, oncogenic, and metabolic pathways, and a worse survival prognosis. Nonetheless, the Immunity-L subtype showed higher ITH and an abundance of angiogenic, neuroactive ligand-receptor interaction, and PPAR pathways relative to the Immunity-H subtype. The previous studies showing the less aggressive nature of angiogenic tumors compared to strongly pro-inflammatory tumors are supported by the higher levels of angiogenesis signature observed in the Immunity-L subtype (39).

A noteworthy observation indicates that Immunity-H, which is distinguished by its high TILs enrichment, has inferior clinical outcomes compared to Immunity-L, which is identified by its low TILs enrichment. The study indicates that “hot” KIRCs carry a less favorable prognosis than “cold” KIRCs, despite the former having a higher enrichment of immune signatures. A similar observation has been reported in other cancer types, such as prostate cancer (40) and gliomas (41). In various cancer types, such as gastric cancer (42), melanoma (43), head and neck squamous cell cancer (44), and triple-negative breast cancer (45), “hot” tumors have a more favorable prognosis than “cold” tumors. The TILs’ enrichment in KIRC has been found to be inversely associated with survival prognosis, and this can be attributed to the inflammation that TILs promote, which in turn promotes tumor progression (46). Additionally, the existence of a noticeably larger percentage of exhausted CD8+ T cells in Immunity-H KIRCs contributes to a certain degree of inadequate antitumor action of TILs in this category, despite its strong TILs enrichment. Furthermore, a previous investigation (26) revealed that the prevalence of exhausted CD8+ cells is linked to an unfavorable prognosis in KIRC.

BAP1 (BRCA1-associated protein 1), a tumor suppressor gene, is involved in regulating the cell cycle and response to DNA damage (47). Our research showed that the mutation rate of BAP1 was higher in Immunity-H than in Immunity-L KIRCs. It is plausible that this could be responsible for the higher genomic instability, proliferation potential, and cell cycle activity of Immunity-H KIRCs in comparison to Immunity-L KIRCs. Furthermore, a previous investigation revealed that the loss of BAP1 caused KIRC cells to be more sensitive to Olaparib, a PARP inhibitor (48). Therefore, considering the elevated HRD level, recurrent BAP1 mutations, and elevated PARP1 expression levels in the Immunity-H subtype, we contend that the therapeutic effectiveness of PARP inhibitors targeting DNA damage repair could be enhanced in this subtype. However, the use of VEGF inhibitors might be proposed as a treatment option for Immunity-L patients given the considerable angiogenic signature observed in this subtype. Moreover, as a result of the heightened TILs and PD-L1 expression levels in Immunity-H KIRCs compared to Immunity-L KIRCs, it can be inferred that patients with Immunity-H will be more responsive to anti-PD-1/PD-L1/CTLA-4 immunotherapy in comparison to those with Immunity-L. In two KIRC cohorts, namely Miao-CM009 (49) and Motzer-CM010 (50), treated with the PD-1 inhibitor Nabumab, it was observed that Immunity-L patients had higher response rates to Nabumab in comparison to Immunity-H patients. The response rates for Immunity-L and Immunity-H patients were 70.6% versus 50.0%, and 66.7% versus 45.8%, respectively. The potential explanation behind these unexpected findings could be the significant depletion of CD8+ T cell function in Immunity-H KIRCs. Despite this, the response rates for the PD-1 inhibitor are relatively high for both Immunity-H and Immunity-L patients when compared to the response rate of less than 15% of cancer patients to ICB up to this point (51). Moreover, understanding the underlying mechanism that induces CD8+ T cell exhaustion in the tumor microenvironment is vital to improve the response to immunotherapy in Immunity-H patients.

It is worth noting that the proportion of female patients in the Immunity-L subtype was significantly higher than in the Immunity-H subtype (42.0% versus 26.7%). On the contrary, the proportion of male patients in the Immunity-L subtype was significantly lower than in the Immunity-H subtype (58.0% versus 73.3%) in TCGA-KIRC. This was established through Fisher’s test with a *P*-value of 0.0002. The results indicate a notable correlation between gender and phenotypic and clinical characteristics of KIRC. There is a wealth of evidence (52) that supports the notion that male patients diagnosed with kidney cancer have more unfavorable prognoses than their female counterparts, which is consistent with our results. In addition, the TCGA-KIRC cohort was composed of 87.7% White, 10.8% African American, and 1.5% Asian populations; the population distribution showed no significant correlation with the immune subtypes (Fisher’s test, *P* = 0.093).

Prior studies have performed molecular classification of KIRC based on gene expressions. For example, Brannon et al. identified two subtypes (ccA and ccB) of KIRC based on the expression profiles of a small gene set by unsupervised consensus clustering (53); ccA was enriched in angiogenesis and displayed a significantly higher survival rate than ccB, and ccB overexpressed cell cycle, response to wounding, and Wnt pathways. It is consistent with our results: (1) Immunity-L KIRCs highly expressed the angiogenesis signature and had a more favorable prognosis; and (2) Immunity-H KIRCs overexpressed cell cycle, immune, and oncogenic pathways and had a less favorable prognosis. Puzanov uncovered three subtypes of KIRC in the TCGA-KIRC cohort and revealed key genetic features of these subtypes (54); consistent with our findings, the aggressive subtype had a higher level of immune infiltration and worse OS than other subtypes. Li et al. classified KIRC into the high-risk group and the low-risk group using a prognostic model built based on the expressions of four metabolic genes (P4HA3, ETNK2, PAFAH2, and ALAD) (55); the high-risk group displayed higher abundance of TILs, in agreement with our results. Additionally, based on the expressions of 279 coagulation-related genes, Yin et al. detected two clusters of KIRC in TCGA-KIRC by consensus clustering (56); among both subtypes, Cluster 2 displayed higher levels of T-cell infiltration and worse survival than Cluster 1. Again, this result is consistent with our findings. A shared finding between these prior investigations and this study is that the KIRC subtype with strong immune infiltration has inferior survival. Nevertheless, our investigation reveals marked discrepancies from the earlier studies. First, it is essential to note that our KIRC clustering involved transcriptomic profiles. However, unlike previous studies (53–56), our approach utilized gene set enrichment analysis rather than individual gene expressions. Undoubtedly, the gene set enrichment-based clustering is more likely to be robust in identifying cancer subtypes than gene expression-based clustering. This is because the gene set-based approach integrates the expression of a set of genes into a single expression value to overcome the vulnerability to expression outliers of individual genes (57). Second, the present study investigated the correlation between tumor immunity and several molecular and clinical features, including tumor stemness, proliferation potential, angiogenesis, microenvironment, genome instability, intratumor heterogeneity, pathway enrichment, and clinical outcomes in KIRC. Ultimately, this study revealed the potential mechanism that underlies the negative association between the enrichment of TILs and clinical outcomes in KIRC.

Moreover, there are several limitations associated with this study. Our discoveries were predominantly made *via* transcriptome data analysis and were not validated at the protein level. The inverse correlation between the TILs enrichment and survival prognosis in KIRC may be attributed to the cellular composition of the tumor microenvironment, including immune cells, stromal cells, and cancer-associated fibroblasts. Verification through experiments is necessary to establish the reliability of certain findings obtained through bioinformatics analysis.

**Figure 6:**
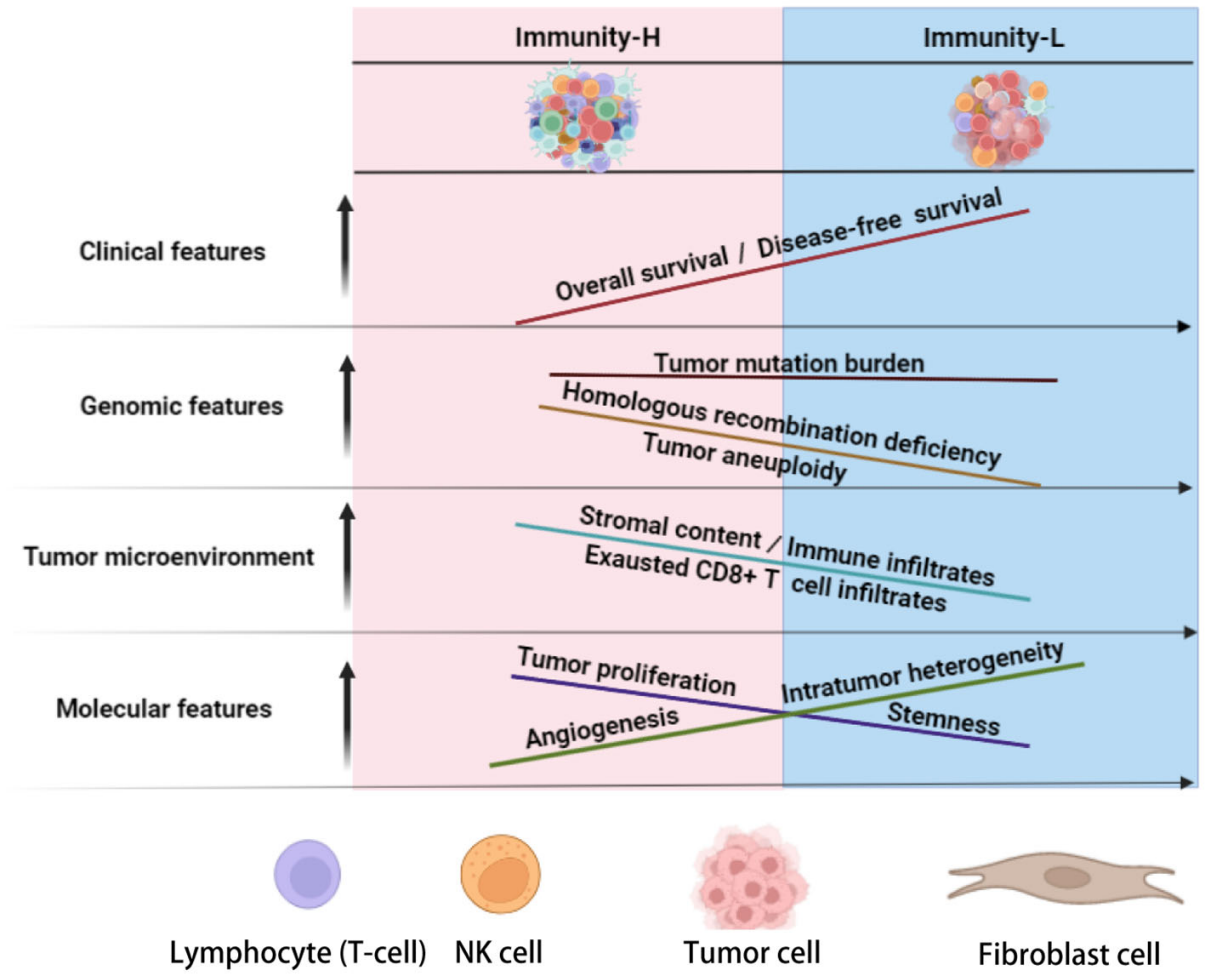
Schematic comparisons of clinical and molecular characteristics between the immune subtypes of KIRC. The figure was created with BioRender.com.

In conclusion, KIRC can be divided into two immune subtypes, each of which exhibits significantly different molecular and clinical characteristics (as depicted in **Figure 6**). Elevated immunity is correlated with a worse prognosis in KIRC. Patients with Immunity-H KIRC could demonstrate active responses to PPAR inhibitors and immune checkpoint inhibitors, whereas those with Immunity-L might exhibit favorable responses to anti-angiogenic agents and immune checkpoint inhibitors. The immunological classification presents molecular insights into KIRC immunity and significant clinical implications for disease management.

## Data availability statement

The original contributions presented in the study are included in the article/**Supplementary Material**. Further inquiries can be directed to the corresponding authors.

## Author contributions

ZC: Software, Validation, Formal analysis, Investigation, Data curation, Writing - review and editing. WC: Software, Validation, Formal analysis, Investigation, Data curation, Visualization, Writing - original draft. JL: Software, Validation, Formal analysis, Investigation, Data curation, Visualization, Writing - original draft. ZA: Investigation, Writing - review and editing. QL: Formal analysis, Investigation. HW: Investigation, Supervision, Project administration. XW: Conceptualization, Methodology, Resources, Investigation, Writing - original draft, Writing - review and editing, Supervision, Project administration. All authors contributed to the article and approved the submitted version.
